# Apolipoprotein A1/C3/A5 haplotypes and serum lipid levels

**DOI:** 10.1186/1476-511X-10-140

**Published:** 2011-08-19

**Authors:** Rui-Xing Yin, Yi-Yang Li, Chao-Qiang Lai

**Affiliations:** 1 Department of Cardiology, Institute of Cardiovascular Diseases, the First Affiliated Hospital, Guangxi Medical University, 22 Shuangyong Road, Nanning 530021, Guangxi, People's Republic of China; 2 Department of Cardiology, Guangxi National Hospital, Nanning 530001, Guangxi, People's Republic of China; 3 Nutrition and Genomics Laboratory, Jean Mayer USDA HNRCA at Tufts University, Boston, MA 02111-1524, USA

## Abstract

**Background:**

The association of single nucleotide polymorphisms (SNPs) in the apolipoprotein (Apo) A1/C3/A4/A5 gene cluster and serum lipid profiles is inconsistent. The present study was undertaken to detect the association between the ApoA1/C3/A5 gene polymorphisms and their haplotypes with serum lipid levels in the general Chinese population.

**Methods:**

A total of 1030 unrelated subjects (492 males and 538 females) aged 15-89 were randomly selected from our previous stratified randomized cluster samples. Genotyping of the ApoA1 -75 bp G>A, ApoC3 3238C>G, ApoA5 -1131T>C, ApoA5 c.553G>T and ApoA5 c.457G>A was performed by polymerse chain reaction and restriction fragment length polymorphism combined with gel electrophoresis, and then confirmed by direct sequencing. Pair-wise linkage disequilibria and haplotype analysis among the five SNPs were estimated.

**Results:**

The levels of high-density lipoprotein cholesterol (HDL-C) and ApoA1 were lower in males than in femailes (*P *< 0.05 for each). The allelic and genotypic frequencies of the SNPs were no significant difference between males and females except ApoC3 3238C>G. There were 11 haplotypes with a frequency >1% identified in the cluster in our population. At the global level, the haplotypes comprised of all five SNPs were significantly associated with all seven lipid traits. In particular, haplotype G-G-C-C-A (6%; in the order of ApoA5 c.553G>T, ApoA5 c.457G>A, ApoA5 -1131T>C, ApoC3 3238C>G, and ApoA1 -75bp G>A) and G-A-T-C-G (4%) showed consistent association with total cholesterol (TC), low-density lipoprotein cholesterol (LDL-C), ApoA1, ApoB, and the ApoA1/ApoB ratio. In addition, carriers of haplotype G-G-T-C-G (26%) had increased serum concentration of HDL-C and ApoA1, whereas carriers of G-G-C-G-G (15%) had high concentrations of TC, triglyceride (TG) and ApoB. We also found that haplotypes with five SNPs explain much more serum lipid variation than any single SNP alone, especially for TG (4.4% for haplotype vs. 2.4% for -1131T>C max based on R-square) and HDL-C (5.1% for haplotype vs. 0.9% for c.553G>T based on R-square). Serum lipid parameters were also correlated with genotypes and several environment factors.

**Conclusions:**

Several common SNPs and their haplotypes in the ApoA1/C3/A5 gene cluster are closely associated with modifications of serum lipid parameters in the general Chinese population.

## Introduction

Dyslipidemia is a common metabolic disorder that may result from abnormalities in the synthesis, processing and catabolism of lipoprotein particles. Elevated plasma total cholesterol (TC) [1], triglyceride (TG) [2], low-density lipoprotein cholesterol (LDL-C) [3], and apolipoprotein (Apo) B [4], together with decreased levels of ApoA1 [4] and high-density lipoprotein cholesterol (HDL-C) [5] are associated with an increased risk of coronary artery disease (CAD). A number of epidemiological studies have shown that in addition to environmental factors, genetic mechanisms may play a role in determining susceptibility to dyslipidemia [6]. The heritability estimates of the interindividual variation in plasma lipid phenotypes from both twin and family studies are in the range of 40-60% [7], suggesting a considerable genetic contribution, and discovery of the genes that contribute to these changes may lead to a better understanding of these processes.

ApoA1 is the predominant protein component of HDL-C, and is involved in the activation of lecithin: cholesterol acyltransferase, which mediate the reverse cholesterol transport from peripheral tissues to the liver [8]. ApoC3 is a major component of TG-rich lipoproteins (chylomicrons and very low density lipoprotein) and a minor component of HDL. The mature 79-amino-acid ApoC3 protein is synthesized predominantly in the liver but also to a lesser extent in the intestine. *In vitro *studies have indicated that ApoC3 is a noncompetitive inhibitor of lipoprotein lipase, thereby suggesting an important role in the catabolism of TG-rich lipoproteins [9]. ApoA5 is a secreted protein present in human serum and is associated with specific lipoprotein particles. It was detectable in very low-density lipoprotein, HDL, and chylomicrons. Serum ApoA5 is very low compared with other apolipoproteins [10]. Human ApoA1/C3/A5 gene resides in the ApoA1/C3/A4/A5 gene cluster, a short region on chromosome 11q23-q24 [11,12]. The ApoA1/C3/A4/A5 gene cluster has been identified as a candidate region for hyperlipidemia in particular for hypertriglyceridemia and atherosclerosis [12,13]. The association between single nucleotide polymorphisms (SNPs) in the ApoA1/C3/A4/A5 gene cluster and plasma or serum lipid levels in humans has been evaluated in a large number of studies [13-30]. However, previous findings on the association of the SNPs in this gene cluster with the changes in plasma lipid levels are inconsistent [31-38]. Many of previous studies for this gene cluster come from studies of hyperlipidemic subjects [14,17,18,25,26,29,33,38] and CAD patients [13,19,21,23,34,36,37]. Studies of differences in the association between ApoA1/C3/A5 gene polymorphisms and serum lipid levels in normolipidemic men and women are extremely limited. Therefore, the aim of the present study was to detect the polymorphisms of ApoA1 -75 bp G>A (rs1799837), ApoC3 3238C>G (rs5128), ApoA5 -1131T>C (rs662799), ApoA5 c.553G>T (rs2075291) and ApoA5 c.457G>A (rs3135507) and the association of their haplotypes and serum lipid levels in the general Chinese population.

## Materials and methods

### Study populations

A total of 1030 unrelated subjects who reside in 16 villages in Napo County, Guangxi Zhuang Autonomous Region, People's Republic of China were randomly selected from our previous stratified randomized cluster samples [39]. The age of the subjects ranged from 15 to 89 years, with an average age of 43.30 ± 17.69 years. There were 492 males and 538 females. All of the subjects were rural agricultural workers. The subjects had no evidence of diseases related to atherosclerosis, CAD and diabetes. None of them had been treated with β-adrenergic blocking agents and lipid-lowering drugs such as statins or fibrates. The present study was approved by the Ethics Committee of the First Affiliated Hospital, Guangxi Medical University. Informed consent was obtained from all subjects after they received a full explanation of the study.

### Epidemiological survey

The survey was carried out using internationally standardized methods. Information on demographics, socioeconomic status, and lifestyle factors was collected with standardized questionnaires. The alcohol information included questions about the number of liangs (about 50 g) of rice wine, corn wine, rum, beer, or liquor consumed during the preceding 12 months. Alcohol consumption was categorized into groups of grams of alcohol per day: < 25 and ≥ 25. Smoking status was categorized into groups of cigarettes per day: < 20 and ≥ 20. At the physical examination, several parameters, such as height, weight, and waist circumference were measured. Sitting blood pressure was measured three times with the use of a mercury sphygmomanometer after the subjects had a 5-minute rest, and the average of the three measurements was used for the level of blood pressure. Systolic blood pressure was determined by the first Korotkoff sound, and diastolic blood pressure by the fifth Korotkoff sound. Body weight, to the nearest 50 grams, was measured using a portable balance scale. Subjects were weighed without shoes and in a minimum of clothing. Height was measured, to the nearest 0.5 cm, using a portable steel measuring device. From these two measurements body mass index (BMI, kg/m^2^) was calculated.

### Biochemical analysis

A venous blood sample of 8 mL was obtained from all subjects after at least 12 hours of fasting. A part of the sample (3 mL) was collected into glass tubes and used to determine serum lipid levels. Another part of the sample (5 mL) was transferred to tubes with anticoagulate solution (4.80 g/L citric acid, 14.70 g/L glucose, and 13.20 g/L tri-sodium citrate) and used to extract deoxyribonucleic acid (DNA). The levels of serum TC, TG, HDL-C, and LDL-C in samples were determined by enzymatic methods with commercially available kits (RANDOX Laboratories Ltd., Ardmore, Diamond Road, Crumlin Co. Antrim, United Kingdom, BT29 4QY; Daiichi Pure Chemicals Co., Ltd., Tokyo, Japan). Serum ApoA1 and ApoB levels were detected by the immunoturbidimetric immunoassay using a commercial kit (RANDOX Laboratories Ltd.). All determinations were performed with an autoanalyzer (Type 7170A; Hitachi Ltd., Tokyo, Japan) in the Clinical Science Experiment Center of the First Affiliated Hospital, Guangxi Medical University [39].

### DNA preparation and genotyping

Total genomic DNA was isolated from peripheral blood leukocytes using the phenol-chloroform method [8,40]. The extracted DNA was stored at 4°C until analysis. Genotyping of the five SNPs was performed by polymerase chain reaction and restriction fragment length polymorphism (PCR-RFLP) [6,8,40]. The sequences of the forward and backward primers used for ApoA1 -75 bp G>A, ApoC3 3238C>G, and ApoA5 -1131T>C were 5'-CACCCGGGAGACCTGCAAGC-3' and 5'-TCTAAGCAGCCAGCTCTTGCA-3', 5'-CACTAGCCCAGAGAGAGGAGTGCC-3' and 5'-CTGAGCCCAGCCGCACACTAA-3', and 5'-GATTGATTCAAGATGCATTTAGGAC-3' and 5'-CCCCAGGAACTGGAGCGAAATT-3' (Sangon, Shanghai, People's Republic of China); respectively. Both ApoA5 c.553G>T and ApoA5 c.457G>A are naturally occurring restriction enzyme sites in the exon 4 of ApoA5 gene. To analyze these two polymorphic markers, exon 4 was amplified using primers 5'-TCGGCGTATGGGTGGAAGAG-3' and 5'-GGCAGCAACTGAAGCCCTACAC-3'. Each reaction system of a total volume of 25 μL, comprised 100 ng (3 μL) of genomic DNA; 0.8 μL of each primer (20 pmo1); 4 μL of 10 × buffer solution; 3 μL dNTP; and 0.4 μL (1 U) *Taq *polymerase. For the amplification, initial denaturation at 95°C for 5 min was followed by 30 cycles of denaturation at 95°C for 15 s, annealing at 61°C for 1 min, and extension at 72°C for 1 min, with final extension at 72°C for 7 min. Each restriction enzyme reaction was performed with 15 μL of amplified DNA; 2 μL of 10 × buffer solution; and restriction ezyme (0.15 μL or 1.5 U *Msp*I for ApoA1 -75bp G>A, 0.2 U *Sst*I for ApoC3 3238C>G, 3 U *Tru1*I or *Mse*I for ApoA5 -1131T>C, 4 U *Msp*I for ApoA5 c.553G>T, and 4 U *Nsb*I or *Fsp*I for ApoA5 c.457G>A) in a total volume of 20 μL digested at 37°C overnight. The digestive products were separated by electrophoresis on sepharose gel. The length of each digested DNA fragment was determined by comparing migration of a sample with that of standard DNA marker. Stained with ethidium bromide, the gel was visualized under ultraviolet light and photographed. Genotypes were scored by an experienced reader blinded to epidemiological data and serum lipid levels.

### DNA sequencing

Twenty-eight samples (each genotype in two) detected by the PCR-RFLP were also confirmed by direct sequencing. The PCR products were purified by low melting point gel electrophoresis and phenol extraction, and then the DNA sequences were analyzed by using an ABI Prism 3100 (Applied Biosyatems) in Shanghai Sangon Biological Engineering Technology & Services Co., Ltd., People's Republic of China.

### Diagnostic criteria

The normal values of serum TC, TG, HDL-C, LDL-C, ApoA1, ApoB levels and the ratio of ApoA1 to ApoB in our Clinical Science Experiment Center were 3.10-5.17, 0.56-1.70, 0.91-1.81, 1.70-3.20 mmol/L, 1.00-1.76, 0.63-1.14 g/L, and 1.00-2.50; respectively [39]. The individuals with TC>5.17 mmol/L and/or TG>1.70 mmol/L were defined as hyperlipidemic [39]. Hypertension was diagnosed according to the criteria of 1999 World Health Organization-International Society of Hypertension Guidelines for the management of hypertension [41]. The diagnostic criteria of overweight and obesity were according to the Cooperative Meta-analysis Group of China Obesity Task Force. Normal weight, overweight and obesity were defined as a BMI < 24, 24-28, and>28 kg/m^2^; respectively [39-41].

### Statistical analysis

Epidemiological data were recorded on a pre-designed form and managed with Excel software. The statistical analyses were done with the statistical software package SPSS 13.0 (SPSS Inc., Chicago, Illinois) or SAS 9.1 (SAS Institute, Inc., Cary, North Carolina, USA). Quantitative variables were expressed as mean ± standard deviation (serum TG levels were presented as medians and interquartile ranges). Qualitative variables were expressed as percentages. Allele frequency was determined via direct counting, and the standard goodness-of-fit test was used to test the Hardy-Weinberg equilibrium. Difference in genotype distribution between the groups was obtained using the chi-square test. The difference in general characteristics between males and females was tested by the Student's unpaired *t*-test. The association of genotypes and serum lipid parameters was tested by analysis of covariance (ANCOVA). Age, BMI, blood pressure, alcohol consumption, and cigarette smoking were adjusted for the statistical analysis. Pair-wise linkage disequilibria (LD) among the five SNPs were estimated as correlation coefficients using the HelixTree program (GOLDEN Helix, Bozeman, MN, USA). For haplotype analysis, we estimated haplotype frequencies using the expectation-maximization algorithm, and determine the association between haplotypes and lipid phenotypes using trend regression analysis with the option of composite haplotype estimation implemented in HelixTree. *P *values were further adjusted for multiple tests by a permutation test. The permutation test was conducted by changing the orders of dependant variable randomly against the genotypes (under the null hypothesis - no association between dependant variable and haplotypes). Then haplotype trend regression was conducted based on the same model and a *P *value was recorded. This process was repeated 1000 times. The *P *values of 1000 permutations were sorted in a descending manner. If the observed *P *value is less than or equal to the 950^th ^*P *value, the association was considered statistically significant. In order to evaluate the association of serum lipid levels (TC ≤ 5.17 mmol/L = 1,>5.17 mmol/L = 2; TG ≤ 1.70 mmol/L = 1,>1.70 mmol/L = 2; HDL-C < 0.91 mmol/L = 1, ≥ 0.91 mmol/L = 2; LDL-C ≤ 3.20 mmol/L = 1,>3.20 mmol/L = 2; ApoA1 < 1.00 g/L = 1, ≥ 1.00 g/L = 2; ApoB ≤ 1.14 g/L = 1,>1.14 g/L = 2; ApoA1/ApoB < 1.00 = 1, ≥ 1.00 = 2) with genotypes (-75bp G>A: GG = 1, GA = 2, AA = 3; 3238C>G: CC = 1, CG = 2, GG = 3; -1131T>C: TT = 1, TC = 2, CC = 3; c.553G>T: GG = 1, GT = 2; c.457G>A: GG = 1, GA/AA = 2) and several environment factors, unconditional logistic regression analysis with forward stepwise modeling was also performed in the combined population of males and females, males, and females; respectively. A *P *value of less than 0.05 was considered statistically significant.

## Results

### Demographic and biochemical characteristics

The demographic and biochemical characteristics of the participants according to sex are presented in Table 1. The levels of body height, weight, systolic blood pressure, diastolic blood pressure, pulse pressure; the prevalence of hypertension; and the percentages of subjects who consumed alcohol or smoked cigarettes were higher in males than in females (*P *< 0.05-0.001), whereas the levels of BMI, HDL-C and ApoA1 were lower in males than in females (*P *< 0.05 for all). There was no significant difference in the levels of age, TC, TG, LDL-C, ApoB, and the ratio of ApoA1 to ApoB (*P *> 0.05 for all).

**Table 1:** Comparison of general characteristics and serum lipid levels of the participants according to sex

Parameter	Male (n = 492)	Female (n = 538)	*t *(χ^2^)	*P*
Age (years)	43.65 ± 18.35	42.98 ± 17.07	0.607	0.544
Height (cm)	156.69 ± 10.25	147.88 ± 6.36	16.725	0.000
Weight (kg)	53.45 ± 8.99	48.17 ± 7.51	10.260	0.000
Body mass index (kg/m^2^)	21.64 ± 2.31	21.96 ± 2.76	-2.008	0.045
> 24 kg/m^2 ^[n (%)]	58 (11.79)	98 (18.22)	8.260	0.004
Systolic blood pressure (mmHg)	125.28 ± 17.14	120.55 ± 14.80	4.751	0.000
Diastolic blood pressure (mmHg)	77.61 ± 10.46	74.45 ± 9.24	5.147	0.000
Pulse pressure (mmHg)	47.70 ± 12.60	46.14 ± 11.35	2.090	0.037
Hypertensive prevalence [n (%)]	96(19.51)	52(9.67)	20.251	0.000
Cigarette smoking [n (%)]				
Nonsmoker	200(40.6)	506(94.0)		
< 20 cigarettes/day	138(28.1)	30(5.6)		
≥ 20 cigarettes/day	154(31.3)	2(0.4)	348.801	0.000
Alcohol consumption [n (%)]				
Nondrinker	186(37.8)	330(61.4)		
< 25 g/day	192(39.0)	204(37.9)		
≥ 25 g/day	114(23.2)	4(0.7)	141.320	0.000
Total cholesterol (mmol/L)	4.61 ± 1.01	4.69 ± 0.90	-1.344	0.179
Triglycerides (mmol/L)	1.00(0.58)	0.97(0.60)	0.206	0.837
HDL-C (mmol/L)	2.03 ± 0.49	2.09 ± 0.46	-2.027	0.043
LDL-C (mmol/L)	2.36 ± 0.74	2.44 ± 0.66	-1.834	0.067
Apolipoprotein (Apo) A1 (g/L)	1.43 ± 0.17	1.45 ± 0.13	-2.131	0.033
ApoB (g/L)	0.90 ± 0.22	0.92 ± 0.20	-1.528	0.127
ApoA1/ApoB	1.71 ± 0.58	1.66 ± 0.44	1.567	0.118

HDL-C, high-density lipoprotein cholesterol; LDL-C, low-density lipoprotein cholesterol. The value of triglyceride was presented as median (interquartile range). The difference between the two sexes was determined by the Wilcoxon-Mann-Whitney test.

### Results of electrophoresis and genotyping

**Figure 1:**
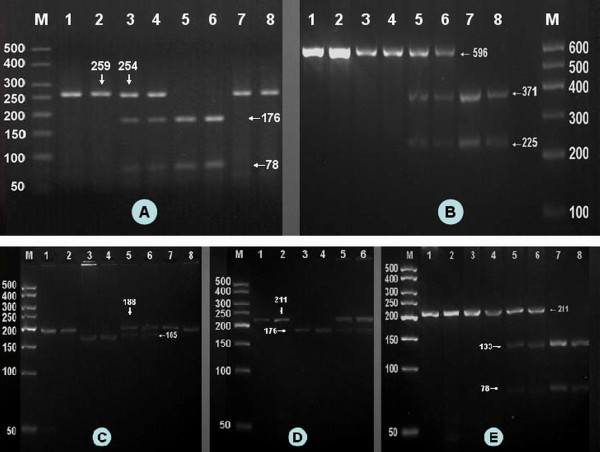
**Genotyping of the ApoA1/C3/A5 gene polymorphisms**. (A) ApoA1 -75 bp G>A. Lane M, 50 bp marker ladder; lanes 1 and 2, the PCR products of the samples (259 bp); lanes 3 and 4, GA genotype (254-, 176- and 78-bp); lanes 5 and 6, GG genotype (176- and 78-bp); and lanes 7 and 8, AA genotype (254 bp). (B) ApoC3 3238C>G. Lane M, 100 bp marker ladder; lanes 1 and 2, the PCR products of the samples (596 bp); lanes 3 and 4, CC genotype (596 bp); lanes 5 and 6, CG genotype (596-, 371- and 225-bp); and lanes 7 and 8, GG genotype (371- and 225-bp). (C) ApoA5 -1131T>C. Lanes 1 and 2, the PCR products of the samples (188 bp); lanes 3 and 4, TT genotype (165- and 23-bp); lanes 5 and 6, TC genotype (188-, 165- and 23-bp); and Lanes 7 and 8, CC genotype (188 bp). The 23 bp fragment was invisible in the gel owing to its fast migration speed. (D) ApoA5 c.553G>T. Lane M, 50 bp marker ladder; lanes 1 and 2, the PCR products of the samples (211 bp); lanes 3 and 4, GG genotype (176- and 35-bp); lanes 5 and 6, GT genotype (211-, 176- and 35-bp). TT genotype was not detected in both sexes. The 35 bp fragment was invisible in the gel owing to its fast migration speed. (E) ApoA5 c.457G>A. Lanes 1 and 2, the PCR products of the samples (211 bp); lanes 3 and 4, GG genotype (211 bp); lanes 5 and 6, GA genotype (211-, 133- and 78-bp); and lanes 7 and 8, AA genotype (133- and 78-bp).

After the genomic DNA of the samples was amplified by PCR and imaged by agarose gel electrophoresis for the ApoA1 -75 bp G>A, the PCR product of 259 bp nucleotide sequences could be seen in the samples. The GG, GA and AA genotypes are shown in Figure 1A. The PCR product of the ApoC3 3238C>G was 596 bp nucleotide sequences. The CC, CG and GG genotypes are shown in Figure 1B. The PCR product of the ApoA5 -1131T>C was 188 bp nucleotide sequences. The TT, TC and CC genotypes are shown in Figure 1C. The PCR product of the ApoA5 c.553G>T was 211 bp nucleotide sequences. The GG and GT genotypes are shown in Figure 1D. The TT genotype was not detected in our study population. The PCR product of the ApoA5 c.457G>A was 211 bp nucleotide sequences. The GG, GA and AA genotypes are shown in Figure 1E.

### Results of sequencing

**Figure 2:**
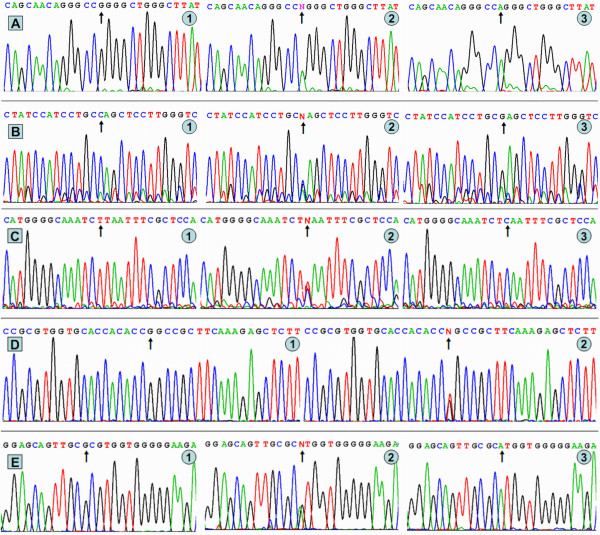
**A part of the nucleotide sequences of the ApoA1/C3/A5 gene polymorphisms**. (A) ApoA1 -75 bp G>A: (1) GG genotype, (2) GA genotype, (3) AA genotype. (B) ApoC3 3238C>G: (1) CC genotype, (2) CG genotype, (3) GG genotype (C) ApoA5 -1131T>C: (1) TT genotype, (2) TC genotype, (3) CC genotype; (D) ApoA5 c.553G>T: (1) GG genotype, (2) GT genotype; (E) ApoA5 c.457G>A: (1) GG genotype, (2) GA genotype, (3) AA genotype.

The results were shown as GG, GA and AA genotypes of the ApoA1 -75bp G>A; CC, CG and GG genotypes of the ApoC3 3238C>G; TT, TC and CC genotypes of the ApoA5 -1131T>C; GG and GT genotypes of the ApoA5 c.553G>T; and GG, GA and AA genotypes of the ApoA5 c.457G>A by PCR-RFLP, the genotypes were also confirmed by sequencing (Figure 2); respectively. We have deposited the raw data at Genbank's Gene Expression Omnibus (GEO) database under accession number GRP3220754.

### Genotypic and allelic frequencies

**Figure 3:**
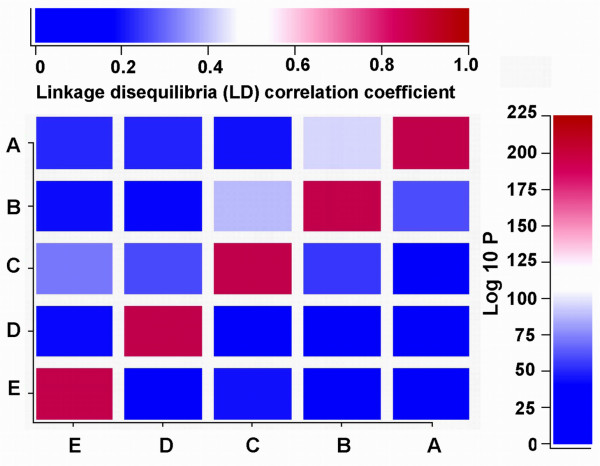
**The linkage disequilibria (LD) among the five SNPs**. (A) ApoA1 -75bp G>A, (B) ApoC3 3238C>G, (C) ApoA5 -1131T>C, (D) ApoA5 c.457G>A, and (E) ApoA5 c.553G>T. ApoA1 -75bp G>A was in LD with ApoC3 3238C>G (LD correlation coefficient *r *= 0.410, *P *< 0.001), ApoA5 -1131T>C (*r *= 0.064, *P *< 0.04), ApoA5 c.553G>T (*r *= 0.106, *P *< 0.001), and ApoA5 c.457G>A (*r *= 0.096, *P *< 0.002). ApoC3 3238C>G was in LD with ApoA5 -1131T>C (*r *= 0.359, *P *< 0.001). ApoA5 -1131T>C was in LD with ApoA5 c.553G>T (*r *= 0.245, *P *< 0.001) and ApoA5 c.457G>A (*r *= 0.165, *P *< 0.001). There was no LD between ApoC3 3238C>G and ApoA5 c.553G>T (*r *= 0.059, *P *= 0.059) or ApoA5 c.457G>A (*r *= 0.044, *P *= 0.155); and between ApoA5 c.553G>T and ApoA5 c.457G>A (*r *= 0.048, *P *= 0.126).

The genotypic frequencies of each of the five loci were all in Hardy-Weinberg equilibrium (ApoA1 -75bp G>A: χ^2 ^= 3.492, *P *= 0.062; ApoC3 3238C>G: χ^2 ^= 0.108, *P *= 0.743; ApoA5 -1131T>C: χ^2 ^= 1.524, *P *= 0.217; ApoA5 c.553G>T: χ^2 ^= 1.200, *P *= 0.273; ApoA5 c.457G>A: χ^2 ^= 2.470, *P *= 0.116; respectively). For five SNPs, ApoA1 -75bp G>A was in LD with ApoC3 3238C>G (*P *< 0.001), ApoA5 -1131T>C (*P *< 0.04), ApoA5 c.553G>T (*P *< 0.001), and ApoA5 c.457G>A (*P *< 0.002). ApoC3 3238C>G was in LD with ApoA5 -1131T>C (*P *< 0.001). ApoA5 -1131T>C was in LD with ApoA5 c.553G>T (*P *< 0.001) and ApoA5 c.457G>A (*P *< 0.001). There was no LD between ApoC3 3238C>G and ApoA5 c.553G>T or ApoA5 c.457G>A; and between ApoA5 c.553G>T and ApoA5 c.457G>A (*P *> 0.05 for all; Figure 3). The frequency of ApoC3 3238C and 3238G alleles was 70.7% and 29.3% in males, and 64.7% and 35.3% in females (*P *< 0.01); respectively. The frequency of CC, CG and GG genotypes was 51.2%, 39.0% and 9.8% in males, and 40.2%, 49.0% and 10.8% in females (*P *< 0.01); respectively. There was no difference in the genotypic and allelic frequencies of the ApoA1 -75bp G>A, ApoA5 -1131T>C, ApoA5 c.553G>T, and ApoA5 c.457G>A between the males and females (*P *> 0.05 for all; Table 2).

**Table 2:** The genotypic and allelic frequencies of ApoA1/C3/A5 gene polymorphisms between males and females

SNP	Group	n	Genotype [n (%)]			Allele [n (%)]	
			
			AA	AB	BB	A	B
ApoA1 -75bp G>A	Male	492	192(39.0)	242(49.2)	58(11.8)	626(63.6)	358(36.4)
(rs1799837)	Female	538	210(39.0)	264(49.1)	64(11.9)	684(63.6)	392(36.4)
	χ^2^	-	0.003			0.001	
	*P*	-	0.998			0.982	
ApoC3 3238C>G	Male	492	252(51.2)	192(39.0)	48(9.8)	696(70.7)	288(29.3)
(rs5128)	Female	538	216(40.2)	264(49.0)	58(10.8)	696(64.7)	380(35.3)
	χ^2^	-	13.053			8.579	
	*P*	-	0.002			0.003	
ApoA5 -1131T>C	Male	492	268(54.4)	178(36.2)	46(9.4)	714(72.6)	270(27.4)
(rs662799)	Female	538	266(49.4)	226(42.0)	46(8.6)	758(70.4)	318(29.6)
	χ^2^	-	3.663			1.127	
	*P*	-	0.160			0.288	
ApoA5 c.553G>T	Male	492	466(94.7)	26(5.3)	0	958(97.4)	26(2.6)
(rs2075291)	Female	538	496(92.2)	42(7.8)	0	1034(96.1)	42(3.9)
	χ^2^	-	2.651			2.561	
	*P*	-	0.103			0.109	
ApoA5 c.457G>A	Male	492	424(86.2)	64(13.0)	4(0.8)	912(92.7)	72(7.3)
(rs3135507)	Female	538	484(90.0)	48(8.9)	6(1.1)	1016(94.4)	60(5.6)
	χ^2^	-	4.605			2.597	
	*P*	-	0.100			0.107	

Allele A: -75bp G, 3238C, -1131T, c.553G or c.457G; Allele B: -75bp A, 3238G, -1131C, c.553T or c.457A; Genotype AA: -75bp GG, 3238CC, -1131TT, c.553GG or c.457GG; Genotype AB: -75bp GA, 3238CG, -1131TC, c.553GT or c.457GA; Genotype BB: -75bp AA, 3238GG, -1131CC, c.553TT or c.457AA

### Genotypes and serum lipid levels

As shown in Table 3, the levels of TG in males were different among the GG, GA and AA genotypes of the ApoA1 -75bp G>A (*P *< 0.003), whereas the levels of HDL-C and the ratio of ApoA1 to ApoB in females were different among the three genotypes (*P *< 0.05 and *P *< 0.01; respectively).

**Table 3:** The ApoA1/C3/A5 genotypes and serum lipid levels between males and females

SNP	Genotype	n	TC (mmol/L)	TG (mmol/L)	HDL-C (mmol/L)	LDL-C (mmol/L)	ApoA1 (g/L)	ApoB (g/L)	ApoA1/ ApoB
ApoA1 -75bp G>A	Male								
(rs1799837)	GG	192	4.57 ± 1.06	0.92(0.46)	2.02 ± 0.46	2.40 ± 0.72	1.42 ± 0.18	0.90 ± 0.22	1.67 ± 0.44
	GA	242	4.67 ± 1.03	1.05(0.70)	2.06 ± 0.54	2.32 ± 0.79	1.45 ± 0.16	0.89 ± 0.23	1.73 ± 0.52
	AA	58	4.51 ± 0.66	0.78(0.54)	1.94 ± 0.37	2.43 ± 0.53	1.41 ± 0.14	0.89 ± 0.21	1.77 ± 1.04
	*F*	-	0.867	11.673	1.458	0.894	2.408	0.117	0.914
	*P*	-	0.421	0.003	0.234	0.410	0.091	0.890	0.402
	Female								
	GG	210	4.73 ± 0.86	0.99(0.62)	2.15 ± 0.51	2.39 ± 0.65	1.46 ± 0.13	0.90 ± 0.21	1.74 ± 0.56
	GA	264	4.68 ± 0.92	0.96(0.59)	2.06 ± 0.42	2.48 ± 0.66	1.45 ± 0.14	0.93 ± 0.20	1.61 ± 0.35
	AA	64	4.61 ± 0.94	0.96(0.59)	2.01 ± 0.47	2.43 ± 0.69	1.42 ± 0.13	0.93 ± 0.21	1.60 ± 0.34
	*F*	-	0.477	0.236	3.273	1.094	2.153	1.369	5.689
	*P*	-	0.621	0.889	0.039	0.336	0.117	0.255	0.004
ApoC3 3238C>G	Male								
(rs5128)	CC	252	4.57 ± 0.96	0.93(0.65)	1.97 ± 0.45	2.34 ± 0.76	1.42 ± 0.15	0.88 ± 0.23	1.75 ± 0.69
	CG	192	4.61 ± 1.02	1.00(0.50)	2.11 ± 0.57^b^	2.35 ± 0.66	1.44 ± 0.19	0.89 ± 0.20	1.68 ± 0.42
	GG	48	4.85 ± 1.21	1.02(0.60)	2.07 ± 0.36	2.57 ± 0.90	1.45 ± 0.15	0.96 ± 0.27	1.64 ± 0.46
	*F*	-	1.551	2.954	4.528	2.036	1.138	2.601	1.213
	*P*	-	0.213	0.228	0.011	0.132	0.321	0.075	0.298
	Female								
	CC	216	4.65 ± 0.80	0.92(0.60)	2.08 ± 0.42	2.45 ± 0.62	1.45 ± 0.12	0.91 ± 0.17	1.64 ± 0.31
	CG	264	4.67 ± 0.98	0.98(0.64)	2.10 ± 0.48	2.41 ± 0.68	1.44 ± 0.14	0.92 ± 0.21	1.67 ± 0.46
	GG	58	4.93 ± 0.87	1.02(1.00)	2.05 ± 0.53	2.56 ± 0.72	1.45 ± 0.12	0.95 ± 0.25	1.68 ± 0.73
	*F*	-	2.342	15.113	0.314	1.249	0.403	0.920	0.339
	*P*	-	0.097	0.001	0.731	0.288	0.668	0.399	0.712
ApoA5 -1131T>C	Male								
(rs662799)	TT	268	4.56 ± 1.01	0.91(0.53)	2.04 ± 0.48	2.33 ± 0.76	1.43 ± 0.15	0.88 ± 0.22	1.76 ± 0.68
	TC	178	4.58 ± 1.01	1.03(0.66)	1.99 ± 0.49	2.34 ± 0.70	1.42 ± 0.19	0.90 ± 0.22	1.67 ± 0.42
	CC	46	5.08 ± 0.90	1.14(0.72)	2.15 ± 0.56	2.66 ± 0.67	1.47 ± 0.12	0.99 ± 0.22	1.57 ± 0.42
	*F*	-	7.637	13.443	0.535	4.492	0.135	8.091	7.797
	*P*	-	0.001	0.001	0.586	0.012	0.873	0.000	0.000
	Female								
	TT	266	4.60 ± 0.84	0.89(0.58)	2.09 ± 0.42	2.39 ± 0.66	1.44 ± 0.13	0.90 ± 0.19	1.69 ± 0.38
	TC	226	4.77 ± 0.99	1.04(0.64)	2.10 ± 0.52	2.49 ± 0.67	1.45 ± 0.14	0.95 ± 0.20	1.61 ± 0.41
	CC	46	4.79 ± 0.77	1.01(1.04)	2.03 ± 0.45	2.48 ± 0.64	1.45 ± 0.12	0.93 ± 0.25	1.74 ± 0.78
	*F*	-	1.662	16.015	0.272	1.233	0.333	2.154	0.278
	*P*	-	0.191	0.000	0.762	0.292	0.717	0.117	0.758
ApoA5 c.553G>T	Male								
(rs2075291)	GG	466	4.61 ± 1.02	0.93(0.56)	2.05 ± 0.50	2.36 ± 0.74	1.43 ± 0.16	0.89 ± 0.22	1.72 ± 0.58
	GT	26	4.64 ± 0.87	1.63(1.39)	1.74 ± 0.33	2.38 ± 0.75	1.39 ± 0.18	0.96 ± 0.27	1.62 ± 0.67
	*F*	-	0.147	4.108	3.122	0.134	1.232	1.559	0.848
	*P*	-	0.883	0.000	0.002	0.893	0.218	0.120	0.397
	Female								
	GG	496	4.70 ± 0.91	0.98(0.60)	2.10 ± 0.47	2.44 ± 0.66	1.45 ± 0.14	0.92 ± 0.20	1.66 ± 0.45
	GT	42	4.60 ± 0.82	0.97(0.74)	1.97 ± 0.33	2.39 ± 0.61	1.45 ± 0.09	0.95 ± 0.21	1.60 ± 0.35
	*F*	-	0.689	0.844	2.359	0.474	0.000	0.930	0.843
	*P*	-	0.491	0.399	0.018	0.636	1.000	0.353	0.399
ApoA5 c.457G>A	Male								
(rs3135507)	GG	424	4.63 ± 0.96	1.00(0.58)	2.07 ± 0.49	2.38 ± 0.74	1.44 ± 0.15	0.90 ± 0.22	1.72 ± 0.59
	GA/AA	68	4.51 ± 1.26	1.01(0.63)	1.79 ± 0.43	2.30 ± 0.73	1.36 ± 0.22	0.88 ± 0.22	1.64 ± 0.50
	*F*	-	0.913	-0.268	4.445	0.829	3.795	0.696	1.059
	*P*	-	0.362	0.788	0.000	0.407	0.000	0.487	0.290
	Female								
	GG	484	4.72 ± 0.91	0.98(0.59)	2.09 ± 0.47	2.47 ± 0.66	1.45 ± 0.14	0.93 ± 0.20	1.64 ± 0.43
	GA/AA	54	4.40 ± 0.81	0.94(0.45)	2.06 ± 0.38	2.21 ± 0.65	1.43 ± 0.10	0.84 ± 0.20	1.82 ± 0.52
	*F*	-	2.477	0.589	0.453	2.750	1.021	3.137	2.452
	*P*	-	0.014	0.556	0.651	0.006	0.309	0.002	0.014

SNP, single nucleotide polymorphism; TC, total cholesterol; TG, triglyceride; HDL-C, high-density lipoprotein cholesterol; LDL-C, low-density lipoprotein cholesterol; ApoA1, apolipoprotein A1; ApoB, apolipoprotein B; ApoA1/ApoB, the ratio of apolipoprotein A1 to apolipoprotein B. The values of TG were presented as median (interquartile range). The difference among the genotypes was determined by the Kruskal-Wallis test or the Wilcoxon-Mann-Whitney test.

The levels of HDL-C in males were different among the CC, CG and GG genotypes of the ApoC3 3238C>G (*P *< 0.05), whereas the levels of TG in females were different among the three genotypes (*P *< 0.01).

The levels of TC, TG, LDL-C, ApoB, and the ratio of ApoA1 to ApoB in males were different among the TT, TC and CC genotypes of the ApoA5 -1131T>C (*P *< 0.05-0.001), whereas the levels of TG in females were different among the three genotypes (*P *< 0.001).

The levels of TG and HDL-C in males were different between the GG and GT genotypes of the ApoA5 c.553G>T (*P *< 0.01 for each), whereas the levels of HDL-C in females were different between the two genotypes (*P *< 0.05).

The levels of HDL-C and ApoA1 in males were different between the GG and GA/AA genotypes of the ApoA5 c.457G>A (*P *< 0.001 for each), whereas the levels of TC, LDL-C, ApoB, and the ratio of ApoA1 to ApoB in females were different between the GG and GA/AA genotypes (*P *< 0.05-0.01).

### Haplotypes and serum lipid levels

To examine the combined effect of five variants (in the order of ApoA5 c.553G>T, ApoA5 c.457G>A, ApoA5 -1131T>C, ApoC3 3238C>G, ApoA1 -75bp G>A) in the cluster, we conducted haplotype analysis with these SNPs on the serum lipid traits (Table 4). There were 11 haplotypes with a frequency>1% identified in the cluster in our population. To increase the statistical power of analysis, we combined five haplotypes with frequencies less than 4% into one group, called "rare-hap", which gives a pooled frequency of 9%. The frequencies of other six haplotypes are listed in Table 4 with G-G-T-C-G and G-G-T-C-A each accounting for 26% haplotype of the population; respectively. At the global level, the haplotypes comprised of all five SNPs were significantly associated with all seven lipid traits (Table 4) with *P *values ranging from 1.56 × 10^-6 ^to 0.001 before or 0.001 to 0.003 after correcting for multiple testing by permuation test. In particular, haplotype G-G-C-C-A (6%) and G-A-T-C-G (4%) showed consistent association with TC, LDL-C, ApoA1, ApoB, and the ratio of ApoA1 to ApoB. In addition, carriers of haplotype G-G-T-C-G (26%) had increased serum concentration of HDL-C and ApoA1, whereas carriers of G-G-C-G-G (15%) had high concentrations of TC, TG, and ApoB.

**Table 4:** Association between ApoA1/C3/A5 haplotypes and serum lipid levels in the combined population of males and females

Lipid	Haplotype	G-G-T-C-G	G-G-T-C-A	G-G-C-G-G	G-G-T-G-G	G-G-C-C-A	G-A-T-C-G	Rare Hap	Haplotype global association	
-	Frequency	0.26	0.26	0.15	0.13	0.06	0.04	0.09	-	-
-	Frequency (carrier vs Noncarrier)	462 vs. 568	458 vs. 572	328 vs. 702	236 vs. 794	118 vs. 912	92 vs. 938	62 vs. 968	*P*	*P *after permutation correction
TC	Carrier	4.67 ± 0.05	4.64 ± 0.05	4.78 ± 0.06	4.71 ± 0.07	4.87 ± 0.09	4.40 ± 0.10	4.70 ± 0.08		
(mmol/L)	Noncarrier	4.70 ± 0.05	4.72 ± 0.05	4.64 ± 0.05	4.68 ± 0.04	4.66 ± 0.04	4.71 ± 0.04	4.69 ± 0.04		
	*P*	0.514	0.159	0.024	0.641	0.021	0.002	0.882	2.05E-04	0.001
TG	Carrier	1.10 (0.07)	1.23 (0.07)	1.38 (0.08)	1.20 (0.09)	1.27 (0.12)	1.73 (0.13)	1.15 (0.16)		
(mmol/L)	Noncarrier	1.33 (0.07)	1.23 (0.07)	1.16 (0.06)	1.24 (0.06)	1.23 (0.06)	1.19 (0.06)	1.24 (0.06)		
	*P*	0.004	0.121	< 0.001	0.866	0.324	0.426	0.055	1.50E-05	0.001
HDL-C	Carrier	2.15 ± 0.03	2.11 ± 0.03	2.14 ± 0.03	2.14 ± 0.04	2.12 ± 0.05	1.95 ± 0.05	2.02 ± 0.04		
(mmol/L)	Noncarrier	2.09 ± 0.03	2.12 ± 0.03	2.10 ± 0.02	2.11 ± 0.02	2.11 ± 0.02	2.13 ± 0.02	2.13 ± 0.02		
	*P*	0.049	0.835	0.279	0.350	0.933	< 0.001	0.014	1.56E-06	0.001
LDL-C	Carrier	2.38 ± 0.04	2.37 ± 0.04	2.45 ± 0.04	2.40 ± 0.05	2.52 ± 0.06	2.14 ± 0.07	2.43 ± 0.06		
(mmol/L)	Noncarrier	2.41 ± 0.04	2.42 ± 0.04	2.37 ± 0.03	2.40 ± 0.03	2.38 ± 0.03	2.42 ± 0.03	2.39 ± 0.03		
	*P*	0.405	0.311	0.085	0.978	0.033	< 0.001	0.561	0.001	0.003
ApoA1	Carrier	1.48 ± 0.01	1.46 ± 0.01	1.46 ± 0.01	1.47 ± 0.01	1.49 ± 0.01	1.41 ± 0.02	1.45 ± 0.01		
(g/L)	Noncarrier	1.46 ± 0.01	1.47 ± 0.01	1.47 ± 0.01	1.46 ± 0.01	1.46 ± 0.01	1.47 ± 0.01	1.47 ± 0.01		
	*P*	0.025	0.581	0.832	0.272	0.043	< 0.001	0.327	6.97E-05	0.001
ApoB	Carrier	0.90 ± 0.01	0.90 ± 0.01	0.93 ± 0.01	0.91 ± 0.02	0.96 ± 0.02	0.83 ± 0.02	0.94 ± 0.02		
(g/L)	Noncarrier	0.92 ± 0.01	0.92 ± 0.01	0.90 ± 0.01	0.91 ± 0.01	0.91 ± 0.01	0.92 ± 0.01	0.91 ± 0.01		
	*P*	0.171	0.207	0.029	0.797	0.008	< 0.001	0.062	6.85E-06	0.001
ApoA1/	Carrier	1.72 ± 0.03	1.72 ± 0.03	1.67 ± 0.03	1.70 ± 0.04	1.59 ± 0.05	1.81 ± 0.06	1.65 ± 0.05		
ApoB	Noncarrier	1.69 ± 0.03	1.69 ± 0.03	1.72 ± 0.03	1.70 ± 0.02	1.72 ± 0.02	1.69 ± 0.02	1.71 ± 0.02		
	*P*	0.357	0.211	0.115	0.975	0.011	0.031	0.173	0.001	0.003

TC, total cholesterol; TG, triglyceride; HDL-C, high-density lipoprotein cholesterol; LDL-C, low-density lipoprotein cholesterol; ApoA1, apolipoprotein A1; ApoB, apolipoprotein B; ApoA1/ApoB, the ratio of apolipoprotein A1 to apolipoprotein B

We also found that haplotypes with five SNPs explain much more serum lipid variation than any single SNP alone, especially for TG (4.4% for haplotype vs 2.4% for -1131T>C max based on R-square) and HDL-C (5.1% for haplotype vs 0.9% for c.553G>T based on R-square).

### Correlation between genotypes and several environment factors and serum lipid levels

For males, multivariate logistic regression analysis showed that the levels of TC were correlated with ApoA5 -1131T>C genotypes; the levels of TG were correlated with ApoA1 -75bp G>A and ApoA5 c.553G>T genotypes, and ApoA5 c.457 G>A, ApoA5 -1131T>C and ApoC3 3238C>G alleles; the levels of LDL-C were correlated with ApoC3 3238C>G genotypes; the levels of ApoB were correlated with ApoA5 c.457 G>A and ApoC3 3238C>G genotypes; the ratio of ApoA1 to ApoB was correlated with ApoA1 -75bp G>A and ApoC3 3238C>G alleles (Table 5).

**Table 5:** Correlation between serum lipid parameters and alleles/genotypes in the males and females

Lipid parameter	Risk factor	χ^2^	*P*	Odds ratio	95% CI
Male plus female					
TC	ApoA5 -1131T>C allele	12.284	0.000	1.704	1.265-2.295
TG	ApoA5 c.553G>T genotype	5.986	0.014	2.293	1.180-4.460
	ApoA5 c.457 G>A allele	7.713	0.005	2.243	1.268-3.968
	ApoA5 -1131T>C allele	6.647	0.010	1.534	1.108-2.123
	ApoA1 -75bp G>A genotype	5.337	0.021	0.345	0.140-0.851
HDL-C	ApoA5 c.457 G>A allele	7.595	0.006	16.571	2.250-12.048
LDL-C	ApoC3 3238C>G genotype	12.001	0.001	2.834	1.572-5.111
ApoA1	ApoA5 c.553G>T genotype	4.978	0.026	11.297	1.343-5.052
ApoB	ApoC3 3238C>G genotype	13.034	0.000	1.514	1.209-1.897
Male					
TC	ApoA5 -1131T>C genotype	4.910	0.027	1.428	1.042-1.957
TG	ApoA5 c.553G>T genotype	17.322	0.000	7.990	3.003-21.260
	ApoA5 c.457 G>A allele	4.097	0.043	2.263	1.026-4.989
	ApoA5 -1131T>C allele	7.400	0.007	2.590	1.305-5.143
	ApoC3 3238C>G allele	3.904	0.048	0.503	0.254-0.995
	ApoA1 -75bp G>A genotype	5.372	0.020	0.240	0.072-0.802
LDL-C	ApoC3 3238C>G genotype	8.307	0.004	1.886	1.225-2.904
ApoB	ApoA5 c.457 G>A genotype	4.600	0.032	0.464	0.230-0.936
	ApoC3 3238C>G genotype	4.284	0.038	2.088	1.040-4.193
ApoA1/ApoB	ApoC3 3238C>G allele	6.836	0.009	0.354	0.163-0.771
	ApoA1 -75bp G>A allele	3.939	0.047	6.065	1.023-35.968
Female					
TC	ApoA5 -1131T>C allele	19.701	0.000	2.651	1.724-4.078
TG	ApoC3 3238C>G genotype	26.171	0.000	3.149	2.029-4.887
ApoB	ApoA5 c.457 G>A allele	7.720	0.005	2.961	1.377-6.368
	ApoA5 -1131T>C genotype	5.390	0.020	1.600	1.076-2.379
	ApoC3 3238C>G allele	14.382	0.000	3.403	1.807-6.407
	ApoA1 -75bp G>A genotype	6.842	0.009	3.043	1.322-7.007
	ApoA1 -75bp G>A allele	4.409	0.036	0.318	0.109-0.927
ApoA1/ApoB	ApoA5 -1131T>C genotype	7.771	0.005	6.339	1.730-23.221
	ApoC3 3238C>G allele	5.542	0.019	13.522	1.546-118.228
	ApoA1 -75bp G>A genotype	5.519	0.019	14.950	1.566-142.771
	ApoA1 -75bp G>A allele	7.989	0.005	0.017	0.001-0.287

TC, total cholesterol; TG, triglyceride; HDL-C, high-density lipoprotein cholesterol; LDL-C, low-density lipoprotein cholesterol; ApoA1, apolipoprotein A1; ApoB, apolipoprotein B; ApoA1/ApoB, the ratio of apolipoprotein A1 to apolipoprotein B

For females, the levels of TC were correlated with ApoA5 -1131T>C alleles; the levels of TG were correlated with ApoC3 3238C>G genotypes, the levels of ApoB were correlated with ApoA1 -75bp G>A and ApoA5 -1131T>C genotypes, ApoA1 -75bp G>A, ApoC3 3238C>G and ApoA5 c.457 G>A alleles; the ratio of ApoA1 to ApoB was correlated with ApoA1 -75bp G>A and ApoA5 -1131T>C genotypes, and ApoA1 -75bp G>A and ApoC3 3238C>G alleles (Table 5).

Serum lipid parameters were also correlated with several environment factors such as age, alcohol consumption, cigarette smoking, blood pressure, body weight, and BMI (Table 6).

**Table 6:** Correlation between serum lipid parameters and several environmental factors in the males and females

Lipid parameter	Risk factor	χ^2^	*P*	Odds ratio	95% CI
Male plus female					
TC	Age	53.062	0.000	1.034	1.025-1.043
	Height	8.532	0.003	0.847	0.757-0.947
	Weight	10.525	0.001	1.329	1.119-1.578
	Body mass index	6.797	0.009	0.593	0.401-0.878
	Cigarette smoking	6.455	0.011	0.748	0.598-0.936
TG	Age	6.792	0.009	1.017	1.004-1.029
	Height	16.601	0.000	0.777	0.688-0.877
	Weight	16.820	0.000	1.476	1.225-1.778
	Body mass index	9.241	0.002	0.522	0.343-0.794
	Alcohol consumption	10.147	0.001	1.606	1.200-2.149
LDL-C	Ethnic group	10.381	0.001	0.492	0.320-0.758
	Sex	5.359	0.021	1.728	1.088-2.747
	Age	28.500	0.000	1.038	1.024-1.052
	Weight	44.497	0.000	1.099	1.069-1.129
	Alcohol consumption	4.813	0.028	0.702	0.511-0.963
ApoA1	Height	13.117	0.000	1.451	1.186-1.775
	Weight	9.403	0.002	0.719	0.583-0.888
	Diastolic blood pressure	7.697	0.006	0.892	0.822-0.967
	Cigarette smoking	6.813	0.009	7.213	1.636-13.806
ApoB	Sex	4.652	0.031	0.665	0.459-0.963
	Height	4.215	0.040	0.903	0.819-0.995
	Weight	5.765	0.016	1.213	1.036-1.419
	Body mass index	4.364	0.037	0.678	0.471-0.976
	Alcohol consumption	5.787	0.016	0.744	0.585-0.947
ApoA1/ApoB	Ethnic group	3.987	0.046	1.752	1.010-3.037
	Height	9.623	0.002	0.815	0.716-0.927
	Weight	12.059	0.001	1.451	1.176-1.791
	Body mass index	14.889	0.000	0.374	0.227-0.616
Male					
TC	Age	5.116	0.024	1.016	1.002-1.031
	Height	5.474	0.019	0.956	0.921-0.993
	Weight	26.172	0.000	1.113	1.068-1.160
TG	Weight	20.023	0.000	1.152	1.083-1.226
	Diastolic blood pressure	4.879	0.027	0.967	0.938-0.996
	Alcohol consumption	14.437	0.000	2.291	1.494-3.513
LDL-C	Ethnic group	5.224	0.022	0.449	0.226-0.892
	Age	8.301	0.004	1.035	1.011-1.060
	Height	14.963	0.000	1.096	1.046-1.148
	Body mass index	22.094	0.000	1.418	1.226-1.640
ApoA1	Height	6.700	0.010	18.279	2.025-5.008
	Weight	6.201	0.013	0.022	0.001-0.442
ApoB	Height	4.635	0.031	0.866	0.760-0.987
	Weight	5.439	0.020	1.285	1.041-1.587
	Body mass index	4.398	0.036	0.576	0.345-0.965
	Systolic blood pressure	9.347	0.002	1.026	1.009-1.044
	Alcohol consumption	4.211	0.040	0.736	0.549-0.986
ApoA1/ApoB	Ethnic group	4.973	0.026	2.286	1.105-4.731
Female					
TC	Age	46.796	0.000	1.051	1.036-1.066
	Weight	6.720	0.010	1.040	1.010-1.071
TG	Age	8.060	0.005	1.028	1.009-1.048
	Height	14.564	0.000	0.648	0.519-0.810
	Weight	13.307	0.000	1.956	1.364-2.805
	Body mass index	9.570	0.002	0.288	0.131-0.643
	Systolic blood pressure	5.469	0.019	1.024	1.004-1.045
HDL-C	Systolic blood pressure	3.389	0.048	0.870	0.758-0.999
LDL-C	Ethnic group	5.274	0.022	0.506	0.283-0.905
	Age	14.902	0.000	1.038	1.018-1.058
	Height	5.072	0.024	1.061	1.008-1.116
	Body mass index	11.305	0.001	1.175	1.070-1.291
ApoA1	Weight	4.470	0.034	0.258	0.074-0.906
	Systolic blood pressure	6.152	0.013	0.583	0.380-0.893
	Pulse pressure	5.363	0.021	1.526	1.067-2.183
ApoB	Age	14.358	0.000	1.032	1.015-1.049
	Weight	5.156	0.023	1.460	1.053-2.023
	Body mass index	4.428	0.035	0.462	0.225-0.948
	Pulse pressure	4.620	0.032	0.975	0.952-0.998
	Cigarette smoking	4.129	0.042	0.303	0.096-0.959
ApoA1/ApoB	Age	6.236	0.013	1.037	1.008-1.067
	Height	9.500	0.002	0.547	0.373-0.803
	Weight	9.508	0.002	2.833	1.461-5.492
	Body mass index	11.423	0.001	0.080	0.019-0.346
	Pulse pressure	5.817	0.016	0.942	0.897-0.989

TC, total cholesterol; TG, triglyceride; HDL-C, high-density lipoprotein cholesterol; LDL-C, low-density lipoprotein cholesterol; ApoA1, apolipoprotein A1; ApoB, apolipoprotein B; ApoA1/ApoB, the ratio of apolipoprotein A1 to apolipoprotein B

## Discussion

The results of the present study clearly show that the levels of serum HDL-C and ApoA1 in the general Chinese population were higher in females than in males. There was no significant difference in the levels of TC, TG, LDL-C, ApoB and the ratio of ApoA1 to ApoB between the two sexes. These findings are in good agreement with those of previous epidemiological studies [42,43]. Although the effects of gonadal hormones on blood lipids are considered contributing factors, the reasons for sex differences in serum lipid levels are still not fully understood. It is commonly accepted that androgens induce changes in lipid concentrations that would predispose towards CAD, whereas estrogens are held to have opposite effects [44,45]. However, much of the evidence for this comes from studies of changes associated with administration of synthetic gonadal steroids or with changes in gonadal function. Studies of differences in lipid metabolism in normal men and women are extremely limited.

There exists significant racial variation of allelic frequencies in this gene cluster, but the present study shows that there was no significant difference in the allelic and genotypic frequencies of the all SNPs except ApoC3 3238C>G between males and females. The frequency of ApoC3 3238G allele was lower in males than in females (29.3% vs. 35.3%, *P *< 0.01). The frequency of CG and GG genotypes was also lower in males than in females (*P *< 0.01). The frequency of ApoA1 -75bp A allele was higher in our population than in Liangshan Yi (23.0%), the other minority in China [46], and Caucasians (12.4-21.8%) in the western countries [47,48]. Rare allelic frequency of Caucasians from different nations was similar and significantly lower than that of oriental races. The frequency of ApoC3 3238G allele in our population was consistent with ranges reported for Chinese (30-43%) [29,49], Japanese (25-48%) [50] and Indians (36%) [51], but are higher than that reported for Caucasians in whom the G allele frequency was 0-11% [51,52]. The ApoA5 -1131C allele in our study population was similar to that in Chinese (29.9%) [53,54], Singaporean Chinese (29.4%) [55], Malays (30.0%) [55], slightly lower than that in Japanese (34.0%) [56,57], but much greater than that of whites (8.0%) [12], Hispanic Americans (16.0%) [16,58,59] or Tunisian (13.0%) [60]. The frequency of ApoA5 c.553T allele in this study is extremely low, and is in agreement with that of two previous studies in Chinese (3.97%) [61] and Chinese Taiwanese (4.2-7.2%) [62,63]. The ApoA5 c.553T allele has been reported to be absent in Caucasians [64]. The ApoA5 c.553TT homozygous was not detected in our study population. This is similar to the results in a previous study [61]. The frequency of ApoA5 c.457A allele was lower in our study population than in Chinese Taiwanese (10.27%) [54], but was higher than that reported for Czechoslovakians in whom the ApoA5 c.457A allele frequency was 2.04% [65]. These results suggest that there exists significant racial/ethinc variation of allelic frequencies in the ApoA1/C3/A5 gene cluster.

The association of the ApoA1/C3/A5 gene polymorphisms and plasma or serum lipid levels in humans has been evaluated in a large number of studies [13-30]. However, previous findings are inconsistent [31-38]. Talmud et al. [66] found that the ApoA1 -75 bp A allele induced elevated HDL-C and ApoA1 levels. This relationship was also demonstrated in many other studies, but with sex-dependent: Jeenah et al. [67] and Sigurdsson et al. [68] found in British and Icelandic male respectively that A allele confered high serum ApoA1 levels. Xu et al. [69] reported A allele carriers had higher mean levels of TC, LDL-C, ApoB and ApoA1 than G homozygotes in Italian boys. Nevertheless, Pagani et al. [70] discovered A allele was positively related to HDL-C concentration in Italian female exclusively. There were also some studies detecting no correlation between the ApoA1 -75 bp G>A and serum lipids pattern [31,32]. Previous cohort studies, as well as case-control and familial studies have shown significant association between the ApoC3 3238G allele and higher plasma TG levels [13-30]. However, several reports failed to find a significant genetic effect on TG concentrations [33-35]. In a previous work, Kee and coworkers found no association between ApoC3 3238C>G polymorphism and lipids, lipoproteins and complex lipoprotein particles in a sample of men from northern France [34]. They thought that the ApoC3 3238C>G polymorphism is not major contributors to the risk of dyslipidemia in the population of northern France. Several separate clinical studies have provided consistent and strong support for the effect with 24% of whites, 35% of blacks, and 53% of Hispanics who carry ApoA5 -1131C allele associated with increased plasma TG levels [12,16,58]. But this association was not significant in a population-based Spanish control group [38]. The ApoA5 c.553G>T polymorphism has been found to correlate strongly with TG levels in Chinese but not in Caucasians [62,64]. The ApoA5 c.553T allele carriers had significantly higher plasma TG levels compared to the wide-type GG genotype, in both CAD and control groups. In a previous study, however, Tang et al. [61] did not find any significant associations between the ApoA5 c.553G>T polymorphism and plasma lipid parameters such as TC, LDL-C, HDL-C, ApoA1, and ApoB in Han Chinese recruited from Jiangsu Province, People's Republic of China. The association of the ApoA5 c.457G>A polymorphism and plasma lipid levels in humans has not been fully elucidated. In a previous study, Kao et al. [62] showed that the ApoA5 c.457G>A polymorphism was not associated with serum TG levels in normal people. In another recent study, Hubacek et al. [65] showed that the impact of statin treatment on lipid parameters did not significantly differ between carriers of the genotypes defined by the ApoA5 c.457G>A polymorphisms. In the present study, we showed that the ApoA5 c.457A allele carriers in males had lower serum HDL-C and ApoA1 levels than the A allele noncarriers, whereas the ApoA5 c.457A allele carriers in females had lower serum TC, LDL-C and ApoB levels and higher the ratio of ApoA1 to ApoB than the A allele noncarriers.

Important intra- and inter-genic LD associations have been found in this study, which replicate previous findings [26-30]. These LD patterns in ApoA1/C3/A5 are rather complex and highly specific to the population under study and indicate the functional dependencies of the encoded proteins [24]. In the present study, haplotype analysis with all five SNPs further supports the strong association between ApoA1/C3/A5 gene polymorphisms and serum lipid levels in our study population. At the global level, the haplotypes comprised of all five SNPs were significantly associated with all seven lipid traits after correcting for multiple testing by permuation test. In particular, haplotype G-G-C-C-A and G-A-T-C-G showed consistent association with LDL-C, TC, ApoA1, ApoB, and the ratio of ApoA1 to ApoB. In addition, carriers of haplotype G-G-T-C-G had increased serum concentration of HDL-C and ApoA1, whereas carriers of G-G-C-G-G had high concentrations of TG, TC, and ApoB. We also found that haplotypes with five SNPs explain much more serum lipid variation than any single SNP alone, especially for TG and HDL-C.

The present study has some shortcomings. Firstly, the size of our study population is a bit small, which might not have had the power to detect the LD across the ApoA1/C3/A5 locus. The individual with ApoA5 c.553TT genotype is not detected in our population, and the number of subjects with ApoA5 c.457AA genotype in both sexes is also small. It has been postulated that an adequate analysis of the polymorphic variants of the ApoA1/ApoC3/ApoA4 gene complex requires a sample of at least 600 subjects to allow the detection of a twofold increased risk of disease [71]. Secondly, the levels of body height, weight, systolic blood pressure, diastolic blood pressure, pulse pressure, the prevalence of hypertension, and the percentages of subjects who consumed alcohol or smoked cigarettes were higher in males than in femailes. Although age, BMI, blood pressure, alcohol consumption, and cigarette smoking have been adjusted for the statistical analysis, we can not completely exclude the influence of these factors on serum lipid levels among different genotypes in both sexes. Thirdly, because we selected the SNPs from literature and did not cover the extensive ApoA1/C3/A4/A5 gene locus, we might miss some information from other SNPs. We did not include ApoA4 SNPs in this study, because there is no ApoA4 SNP is associated with hypertriglyceridemia [29], except ApoA4 T347S associated with a TG-lowering effect [16]. Chien et al. [30] have analyzed amino acid 360 (G to T substitution, Glu to His) and amino acid 347 (A to T, Thr to Ser) of the ApoA4 gene but the results showed no substitution was found in a community-based population.

## Conclusion

The present study shows that the allelic and genotypic frequencies of the all SNPs except ApoC3 3238C>G were not different between males and females, but all five SNPs at the ApoA1/C3/A5 gene cluster and their haplotypes are closely associated with modifications of serum lipid parameters in both sexes. The haplotypes with five SNPs explain much more serum lipid variation than any single SNP alone, especially for TG and HDL-C in the general Chinese population.

## Competing interests

The authors declare that they have no competing interests.

## Authors' contributions

RXY conceived the study, participated in the design, carried out the epidemiological survey, collected the samples, performed statistical analyses, and drafted the manuscript. YYL participated in the design, undertook genotyping, and helped to perform statistical analyses. CQL participated in the design, performed statistical analyses, and helped to draft the manuscript. All authors read and approved the final manuscript.
